# Laparoscopic removal of a broken acupuncture needle in pancreatic head: a case report

**DOI:** 10.1093/jscr/rjae714

**Published:** 2024-11-24

**Authors:** Kil Hwan Kim, Sungho Jo, Sanghyun Song

**Affiliations:** Department of Surgery, Dankook University Hospital, Dankook University College of Medicine, 201 Manghyang-ro, Dongnam-gu, Cheonan, Korea; Department of Surgery, Dankook University Hospital, Dankook University College of Medicine, 201 Manghyang-ro, Dongnam-gu, Cheonan, Korea; Department of Surgery, Dankook University Hospital, Dankook University College of Medicine, 201 Manghyang-ro, Dongnam-gu, Cheonan, Korea

**Keywords:** acupuncture, minimally invasive surgery, back pain, epigastric pain

## Abstract

Acupuncture, a well-established traditional medical practice in East Asia, is rarely associated with complications associated with broken needles. A 45-year-old woman had received acupuncture treatment 3 months before presentation to relieve back pain and complained of persistent epigastric pain. Radiological studies revealed chronic cholecystitis with stones and a broken acupuncture needle in the pancreatic head. Laparoscopic cholecystectomy and foreign body removal were performed, and the patient recovered quickly during a short hospital stay. We confirm that a needle found in the pancreas can usually be safely removed with minimally invasive surgery.

## Introduction

Acupuncture, a well-known traditional medical practice originating in East Asia, is widely used globally to treat various conditions, especially pain. However, multiple complications associated with acupuncture have been documented in the literature [1–4]. Severe adverse events (AEs) have been reported, with previous research indicating that acupuncture leads to 0.5 AEs/10000 procedures [5]. Severe AEs from acupuncture include cardiac tamponade, changes in neurological signs, intra-abdominal organ injury, cerebral hemorrhage, and death.

Injuries to the intraabdominal organs have been reported in some studies; however, cases of pancreatic injury or broken acupuncture needles in the pancreas have not been reported. Here, we report a case of successful laparoscopic removal of a fractured acupuncture needle from the pancreatic head.

## Case report

A 45-year-old woman with diabetes, hypothyroidism, and hepatitis B presented with persistent epigastric pain for 1 month. She mentioned eating corvina a month before the presentation and had been experiencing abdominal discomfort. During physical examination, the patient’s abdomen was thoroughly assessed, and no tenderness was observed.

**Figure 1:**
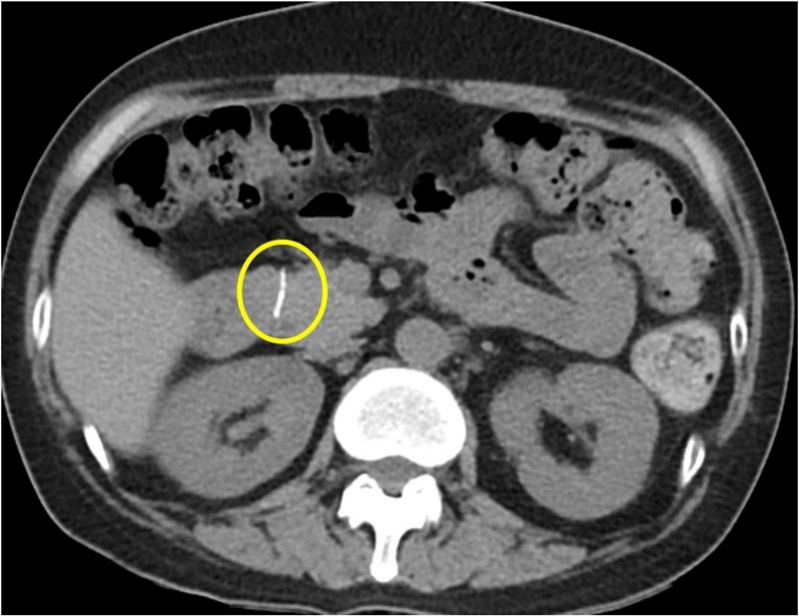
Abdominal CT scan showing a 1.7-cm linear high-density foreign body penetrating the anterior aspect of the head of pancreas.

Initial radiography showed a small, calcified line in the upper abdomen, although its size was too small to be easily overlooked. Laboratory results showed normal liver function and pancreatic enzyme levels, with a white blood cell count of 3290/mm and C-reactive protein level of 0.09 mg/dl. Abdominal CT revealed a 1.7-cm linear high-density foreign body penetrating the anterior aspect of the pancreatic head (Fig. 1). We also identified multiple stones in the collapsed gallbladder with significant wall thickening on CT.

**Figure 2:**
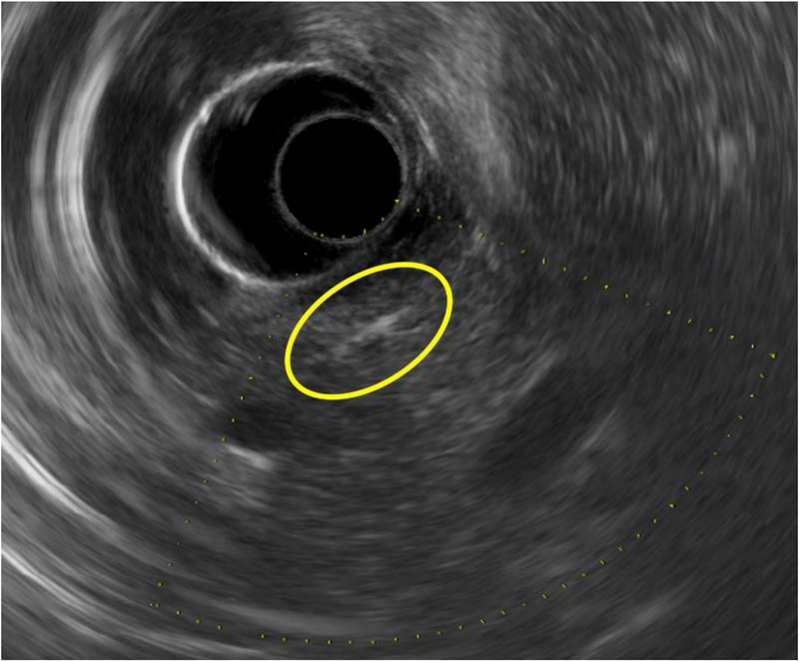
A linear and straight highly echogenic substance is seen in the parenchyma of the pancreatic head on endoscopic ultrasonography.

Gastroduodenoscopy showed normal mucosa of the duodenum without any foreign body. Endoscopic ultrasonography (EUS) detected a linear and straight highly echogenic substance in the parenchyma of the pancreatic head (Fig. 2). Using EUS, we predicted the foreign body to be a needle, not a fish bone.

**Figure 3:**
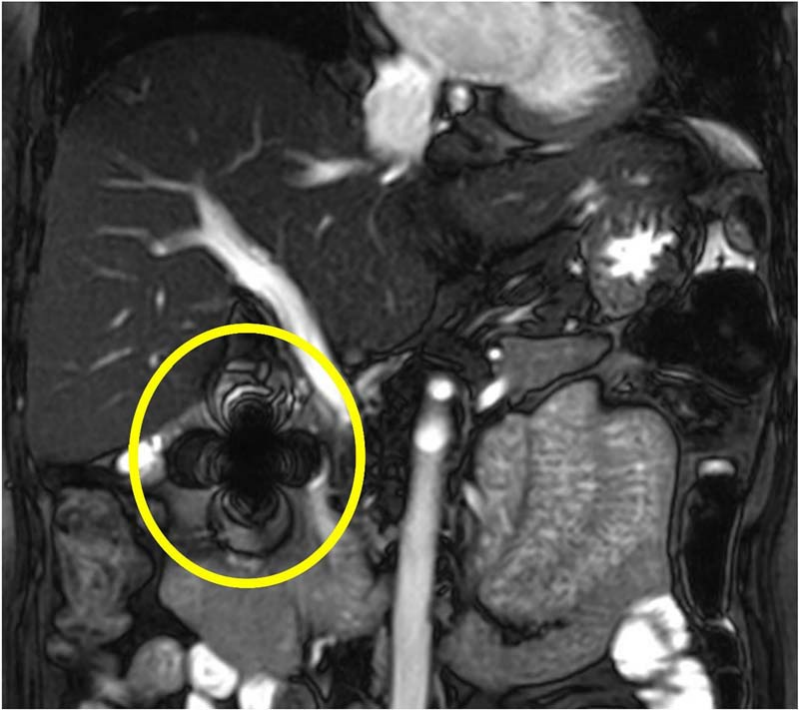
MRCP shows a severe ferromagnetic artifact in the pancreatic head and the second part of the duodenum, suggesting the presence of a metal foreign body in that area.

Magnetic resonance cholangiopancreatography (MRCP) was performed to determine the relationship between the foreign body and the main pancreatic duct. MRCP showed a severe ferromagnetic artifact in the pancreatic head and the second part of the duodenum, suggesting the presence of a metal foreign body in that area (Fig. 3). After MRCP findings, the patient disclosed that she had received acupuncture therapy from an acupuncturist at a Korean Oriental medical clinic for back pain 3 months earlier. She was referred to our department for the surgical treatment of chronic cholecystitis and a pancreatic foreign body. Hence, an elective laparoscopic surgery was performed.

**Figure 4:**
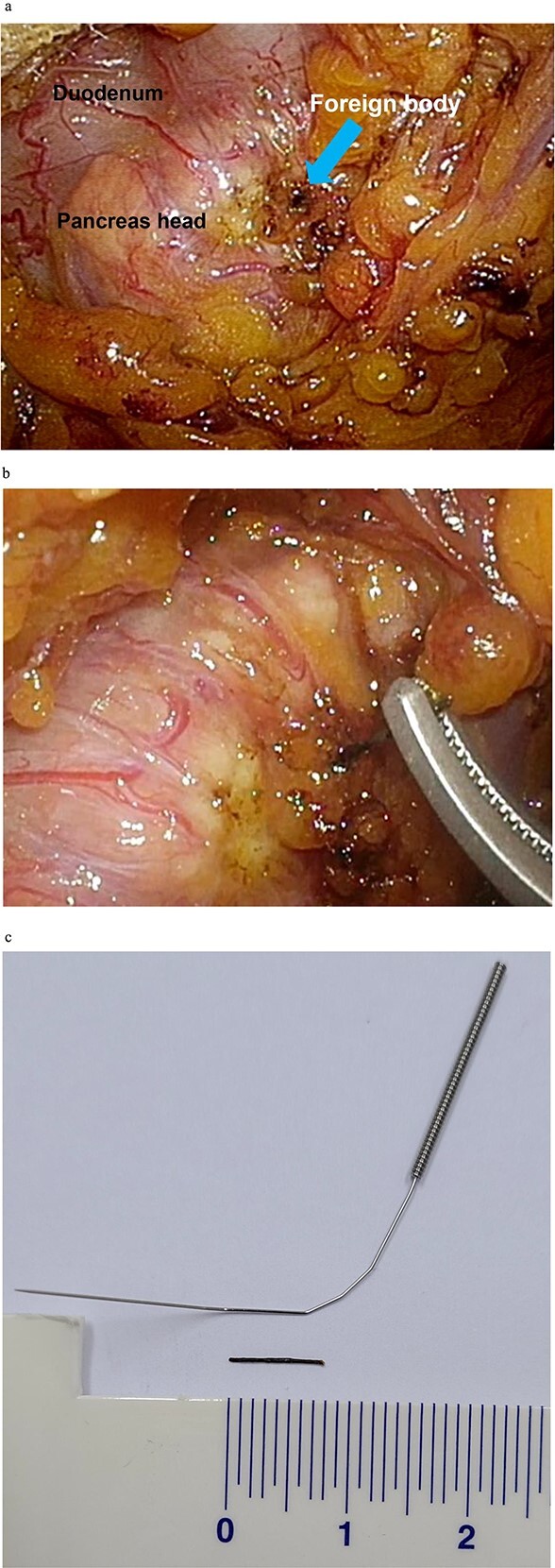
Intraoperative images showing the (a) identification of the foreign body, (b) removal of the foreign body, and (c) comparison of the foreign body with an intact acupuncture needle.

A 3D camera was used, and four trocars were placed: one below the umbilicus for laparoscopy (12 mm), two in the upper right abdominal quadrant (5 mm), and one in the upper left abdominal quadrant (5 mm). Partial laparoscopic transection of the gastrocolic ligament was performed to access the lesser sac. After cranial retraction of the stomach, the anterior aspect of the pancreatic head was identified through further dissection. Initially, locating the foreign body was initially challenging; however, it became identifiable after clearing the area around the pancreatic head. The body was hard to spot initially because the 1 cm-long metal needle turned black and was positioned backward in front of the pancreas, resulting in the appearance of a spot (Fig. 4a–c). After carefully removing the foreign body, we confirmed the absence of pancreatic juice leakage and applied fibrin glue. Subsequently, the conventional cholecystectomy was completed without any significant events. A mixed pigmented stone was lodged in the gallbladder neck, with two additional stones found within the lumen.

External drainage was not installed, and the operation time was 120 min. The final biopsy confirmed the presence of chronic cholecystitis in the gallbladder. The patient recovered without any complications and was discharged on the fourth postoperative day.

## Discussion

Acupuncture, although not widely recognized in Western medicine, is an integral part of the healthcare system in East Asia. The core theory of acupuncture centers on the principle of qi, a vital energy essential for maintaining health and flows throughout the body. Disruptions in the qi flow are believed to compromise the body’s regulatory mechanisms. Acupuncture aims to reestablish equilibrium by targeting specific points near the skin to correct these disturbances [6]. Although it is known to be a relatively simple procedure, adverse effects requiring specific treatments, such as organ, tissue, and nerve injuries, have been reported in up to 2.2% of clinical cases [7].

In a systematic review conducted in China during 1980–2013 [8], seven patients with broken or bent needles were reported. Five patients recovered successfully after surgical intervention, whereas, in the remaining two cases, acupuncturists gradually removed the bent needles. Other systematic reviews in Korea and China (2010–2023) identified 23 cases of broken or retained acupuncture needles [9].

The World Health Organization states that acupuncture needle breakage can occur due to various factors including substandard manufacturing quality, erosion at the shaft–handle juncture, strong muscle spasms, sudden patient movements, incorrect removal of a stuck or bent needle, and prolonged use of galvanic current [10, 11]. There have been reports of acute pancreatitis following acupuncture and cases of pancreatic foreign bodies due to needle or fish bone ingestion; however, no studies have found broken acupuncture needles within the pancreas to date.

In this case, because of the presence of the needle in the anterior aspect of the pancreatic head, it was possible to safely remove it via laparoscopic surgery. The patient had a brief hospital stay and rapidly recovered without postoperative complications after the minimally invasive surgery. If the needle is confined to the retroperitoneum or not visible outside the pancreas, ultrasound may be used for detection; however, removal through laparoscopy might be challenging.

In conclusion, acupuncture is generally safe. However, complications can occur, making it crucial for trained professionals to perform the procedure. Rare cases of broken needles have been reported. If a needle is found in the pancreas, it can usually be safely removed with minimally invasive surgery.
